# *QuickStats*: Age-Adjusted Percentage* of Adults Aged ≥18 Years with Serious Psychological Distress During the Past 30 Days,^†^ by Family Income^§^ — National Health Interview Survey, 2021^¶^

**DOI:** 10.15585/mmwr.mm7212a5

**Published:** 2023-03-24

**Figure:**
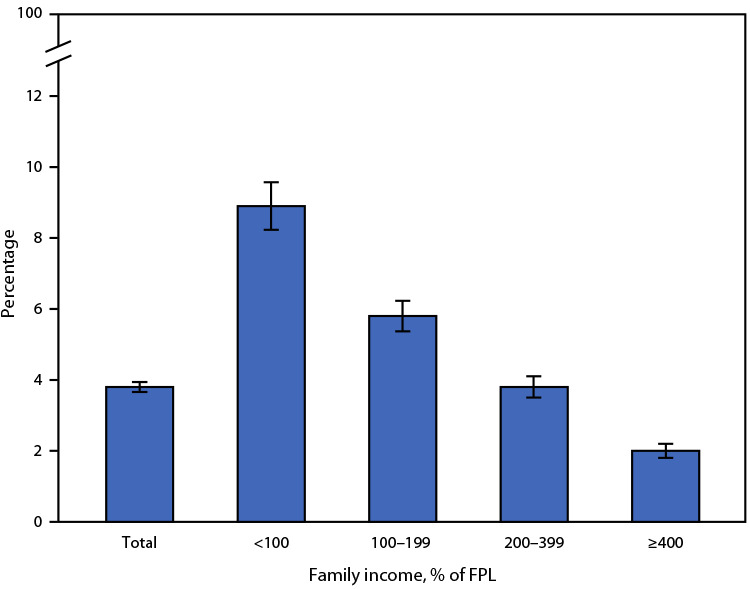
In 2021, 3.8% of adults aged ≥18 years had serious psychological distress during the past 30 days. The age-adjusted percentage of adults who had serious psychological distress decreased with increasing family income, from 8.9% of adults with family income <100% of FPL, to 5.8% of adults with family income 100%–199% of FPL, to 3.8% of adults with family income 200%–399% of FPL, and to 2.0% of adults with family income ≥400% of FPL.

**Source:** National Center for Health Statistics, National Health Interview Survey, 2021. https://www.cdc.gov/nchs/nhis/index.htm

